# Prevalence of asymptomatic malaria and associated factors among pregnant women in Mogadishu, Somalia: a cross-sectional study

**DOI:** 10.1186/s12936-025-05665-3

**Published:** 2025-11-24

**Authors:** Mohamed Yusuf Abdi, Faiso Aweis Mohamud, Suweyda Abdiaziz Abdullahi

**Affiliations:** Department of Microbiology and Laboratory Sciences, Faculty of Medicine and Health Sciences, SIMAD University, Mogadishu, Somalia

**Keywords:** Prevalence, Asymptomatic malaria, Pregnant women, Somalia

## Abstract

**Background:**

Asymptomatic *Plasmodium* infections during pregnancy can cause serious complications such as stillbirths, abortions, premature deliveries, and low birth weight infants. Furthermore, these silent infections hinder malaria control efforts as asymptomatic individuals can unknowingly transmit *Plasmodium* within communities. This study aimed to assess the prevalence of asymptomatic malaria and its associated factors in pregnant women in Mogadishu, Somalia.

**Methods:**

A cross-sectional investigation was conducted at Ayaan Hospital in Mogadishu, Somalia, involving 171 asymptomatic expectant mothers, selected via random sampling between February and March 2025. The data collection methods included questionnaires, rapid diagnostic tests (RDTs), and microscopic examinations of blood samples. The statistical analysis employed descriptive statistics, chi-square test, Fisher’s Exact test, and odds ratios with 95% confidence intervals, with statistical significance established at p < 0.05.

**Results:**

Among the 171 pregnant women evaluated, 2.9% had asymptomatic *Plasmodium* infections, with *P. falciparum* was the only species detected in all positive cases. No significant associations were observed between infection and sociodemographic, obstetric, or malaria—prevention factors. However, younger age (OR = 0.24; 95% CI 0.03–2.23), urban residence (OR = 0.12; 95% CI 0.012–1.32), and ITN utilization (OR = 0.06; 95% CI 0.002–1.82) were associated with lower odds of infection, suggesting potential protective effects.

**Conclusions:**

This study found a low prevalence of asymptomatic *Plasmodium falciparum* infection in pregnant women. While no factors were significantly associated with asymptomatic *Plasmodium* infection, the findings suggest that younger age, urban residence, and ITN utilization may have protective effects. Further research with larger samples is needed to confirm these observations.

## Background

In 2023, Somalia reported an estimated 1,081,187 malaria cases, resulting in approximately 2611 fatalities. Malaria transmission occurs year-round in Somalia, with the predominant species, *Plasmodium falciparum*, responsible for 98% of cases, whereas *Plasmodium vivax* accounts for the remaining 2% [1]. In Mogadishu, the prevalence of malaria shows significant variation, with rates reported to be between 4.3% and 60% among different populations [2–5]. This variability likely reflects challenges such as limited data quality, false negatives, and healthcare access issues exacerbated by conflict and natural disasters. Seasonal, demographic, and spatial factors further complicate surveillance and control efforts [1]. Among these complexities, gestational malaria is a particular public health concern [6].

Pregnant women in endemic regions are at an increased risk of asymptomatic malaria, defined as the presence of *Plasmodium* parasites without clinical symptoms. This condition often goes undiagnosed due to placental sequestration, reduced immune detection, and limited access to healthcare services [7, 8]. Despite the absence of symptoms, asymptomatic malaria can result in serious consequences. In pregnant women, it may lead to maternal anaemia through *Plasmodium*-induced haemolysis, reducing oxygen delivery to both mother and fetus [8–10]. It also increases the risk of preterm birth, low birth weight, and intrauterine growth restriction, all of which contribute to increased neonatal morbidity and mortality [11].

Beyond individual outcomes, asymptomatic pregnant women serve as reservoirs of transmission, complicating malaria elimination efforts [10, 12]. Mitigating the detrimental impact of gestational malaria necessitates the integration of prophylactic measures into prenatal healthcare protocols within endemic regions, notably across sub-Saharan Africa [13]. The World Health Organization (WHO) is currently endeavouring to augment the accessibility of intermittent preventive treatment with sulfadoxine‒pyrimethamine (IPT-SP) administration for expectant mothers in African regions with moderate to elevated malaria transmission rates. This initiative is implemented through incorporation into the existing prenatal healthcare services [14]. However, efforts to eradicate malaria continue to face challenges in achieving the level of effectiveness anticipated by the WHO and key collaborators. National-level data on malaria incidence and associated risk factors are variable. Despite the known risks of asymptomatic malaria during pregnancy, data on its prevalence and associated factors in Somalia, particularly in community settings, remain limited. Addressing this gap is critical for the development of effective strategies for maternal health and malaria control. Thus, this study aimed to determine the prevalence of asymptomatic *Plasmodium* infection among pregnant women in Mogadishu, Somalia, and to identify factors associated with asymptomatic malaria infection in this population.

## Methods

### Study area and period

This study was conducted at the Ayaan Hospital in Mogadishu, Somalia, from February 1 to March 31, 2025. Ayan Hospital is a private, multispecialty institution located in the Banadir region of Mogadishu that serves a densely populated urban community with limited access to public healthcare services. The facility comprises 120 beds and includes departments such as internal medicine, obstetrics and gynaecology, surgery, and paediatrics. In addition, it provides specialized services including dialysis and diagnostic imaging. The hospital predominantly caters to low- to middle-income patients, with a particular focus on women and children, addressing medical conditions such as infections, chronic diseases, and trauma. Ayaan Hospital was selected because it serves a large number of patients and is easily accessible to pregnant women in the community, making it a suitable and practical setting for the present study.

### Study design and population

This hospital-based cross-sectional study included asymptomatic healthy pregnant women who exhibited no signs or symptoms of malaria within the preceding 48-h period and provided informed consent. The exclusion criteria were recent antimalarial use within the 4 weeks preceding the study period, psychiatric conditions, severe comorbidities, chronic therapy, and refusal to participate.

### Sample size determination and sampling technique

The sample size for prevalence estimation was determined using a single-population proportion formula. The calculation incorporated the following parameters: a previously documented 12.7% prevalence of asymptomatic malaria in pregnant women in Burkina Faso [9], a 95% confidence interval, and a 5% margin of error. This computation provided the requisite sample of 171 subjects. The participants were selected using a simple random sampling method.

### Study variables and data collection procedure

The dependent variable in this study was the prevalence of asymptomatic malaria among pregnant women. The independent variables included sociodemographic factors (age, residence, marital status, educational level, and occupational status), obstetric factors (gravidity, gestational trimester, and history of *Plasmodium* infection within the previous year), behavioural factors (antenatal care (ANC) visits, possession and use of insecticide-treated bed nets (ITNs), and indoor residual spraying (IRS) usage), and environmental and clinic-related factors, including proximity to stagnant water, presence of mosquitoes in the household, availability and administration of intermittent preventive treatment during pregnancy (IPTp), and whether participants had received education on malaria prevention methods.

Data were collected using a semi-structured interviewer-administered questionnaire conducted in Somali. Participants’ responses were recorded in accordance with the questionnaire guidelines. Clinical data, including gravidity, trimester, and ANC visits, were cross-checked with antenatal medical records of each participant to ensure accuracy. To maintain data quality, the questionnaire was pre-tested before data collection, and data collectors received training on standard data and sample collection procedures. After the interview, blood sample was collected from each participant using standard aseptic techniques. Malaria was diagnosed using both microscopy and rapid diagnostic tests (RDTs)**.**

### Blood specimen collection

Following the interviews, approximately 2 mL of blood was drawn from the study participants via the peripheral vein into an ethylenediaminetetraacetic acid (EDTA) tube. The samples were then labelled, stored in cool boxes, and transported to the Microbiology Laboratory, Faculty of Medicine and Health Sciences, SIMAD University, Mogadishu, Somalia, for diagnosis.

### Laboratory investigation

To detect *Plasmodium* infections, both rapid diagnostic tests (RDTs) and microscopic examination of stained blood smears are used as diagnostic methods.

### Rapid diagnostic tests (RDTs)

Parasite detection using Malaria Pf/Pv (anti-HRP-II/anti-LDH) Ag Combo RDTs (Hangzhou Yicare Biotech Co., Ltd, China) was conducted according to the manufacturer’s guidelines. A micropipette was used to add ten microliters of blood to the sample well, and three drops (80 μL) of buffer solution were added to the buffer well. After 10–15 min wait, the results were recorded according to the manufacturer’s instructions [15].

### Malaria microscopy

For each participant, two glass slides were used to prepare the thick (6 µL) and thin (2 µL) blood films. The thin film was fixed in absolute methanol for 10–15 s prior to staining with 10% Giemsa stain for 10 min. Subsequently, the films were examined at 1000X magnification using an Olympus CX23 light microscope (Olympus, Japan) by a trained medical laboratory technologist. The diagnostic protocol involved examining blood films to identify *Plasmodium* parasites, determine their species, and assess their developmental stages. The specimens were deemed uninfected if no parasites were observed across the 200 high-power fields [16].

### Operational definitions

- **Asymptomatic malaria** refers to the presence of a malaria infection without the manifestation of clinical signs and symptoms.
- **Gravidity**: The total number of pregnancies the participant had, including the current pregnancy.
- **Trimester**: The stage of gestation during which the data was collected.
- **Previous history of *Plasmodium* infection**: Malaria infection was clinically confirmed in the last year.
- **Antenatal care (ANC) visits**: The number of antenatal appointments attended during the current pregnancy.
- **Possession of insecticide-treated bed net (ITN)**: Whether the participants owned at least one ITN.
- **Use of ITN**: Whether the participant reported sleeping under an ITN the night before the survey.
- **Indoor residual spray (IRS) usage**: Whether participant’s household has received an IRS application in the last year.
- **Proximity to stagnant water**: Whether participant resides within 500 m of a body of stagnant water, such as a pond or swamp, as determined by self-report.
- **Presence of mosquitoes in the household**: The self-reported presence of mosquitoes inside the home.
- **Availability of intermittent preventive treatment in pregnancy (IPTp)**: Whether the healthcare facility that the participant visits consistently offer sulfadoxine-pyrimethamine.
- **Receipt of IPTp**: Whether the participant was administered at least one dose of IPTp during her current pregnancy and confirmed by medical records.
- **Education about malaria prevention**: Whether participant was given any formal instruction or advice on preventing malaria during ANC appointments.

### Data analysis

Data were entered, cleaned, and analysed using SPSS Statistics version 20. Descriptive statistics were used to summarize the characteristics of the study population. The prevalence of asymptomatic *Plasmodium* infections among pregnant women was determined using frequency distributions. To explore the association between asymptomatic malaria and potential risk factors, odds ratios (ORs) with 95% confidence intervals (CIs) were calculated. For categorical variables, statistical significance was assessed using Fisher’s Exact Test, which was chosen because of the small sample size and presence of low expected cell counts in several comparisons. Statistical significance was set at P < 0.05.

## Results

### Sociodemographic and obstetric characteristics of pregnant women in Mogadishu, Somalia

In a survey of 171 pregnant women, nearly half (49.7%) were aged between 18 and 24 years, 39.2% were between 25 and 34 years, and 11.1% were older than 34 years. Most of the participants were married (90.6%) and predominantly resided in urban areas (96.5%). More than half (50.9%) had not received formal education, 18.1% had completed primary education, 25.1% had completed secondary education, and 5.8% had attained a diploma-level education. The majority of participants were unemployed (93%). Regarding obstetric characteristics, 46.2% were in the third trimester, 36.8% in the second trimester, and 17.0% in the first trimester. Most participants (74.9%) had multiple pregnancies. Additionally, 63.2% of participant reported having malaria, indicating prior exposure to the disease (Table 1).

**Table 1:** Sociodemographic and obstetric characteristics of pregnant women in Mogadishu, Somalia

Variables	Frequency	Percent (%)
Age category		
18–24 years	85	49.7
25–34 years	67	39.2
> 34 years	19	11.1
Marital status		
Married	155	90.6
Widowed/Divorced	16	9.4
Residence		
Urban	165	96.5
Rural	6	3.5
Education level		
No formal education	87	50.9
Primary	31	18.1
Secondary	43	25.1
College and above	10	5.8
Occupational status		
Employed	12	7.0
Un-employed	169	93.0
Gestational age		
First trimester	29	17.0
Second trimester	63	36.8
Third trimester	79	46.2
Gravidity status		
One time	43	25.1
More than one time	128	74.9
Previous history of malaria infection		
Yes	108	63.2
No	63	36.8

### Behavioural characteristics related to malaria prevention and antenatal care services among pregnant women in Mogadishu, Somalia

A study of behavioural patterns indicated that 69.1% of pregnant women had never engaged in antenatal care (ANC), whereas only 4.1% had attended ANC more than four times. Insecticide-treated net (ITN) ownership was notably low, with only 16.4% of the women reporting that they owned one. Among those who owned ITNs, a high proportion (82.1%) reported using the net at least once during pregnancy, while 17.9% never used it. Indoor residual spraying (IRS) was relatively well covered, with 72.4% of participants reporting their homes being sprayed. However, the adoption of intermittent preventive treatment with sulfadoxine-pyrimethamine (IPTp-SP) was exceedingly rare: 96.5% had not received any dose, and only 0.6% had received the recommended two doses (Table 2).

**Table 2:** Behavioural characteristics related to malaria prevention and antenatal care services among pregnant women in Mogadishu, Somalia

Variables	Frequency	Percent (%)
ANC follow up		
Never	84	69.1
1–2 times	60	35.1
3 times	20	11.7
> 4	7	4.1
Insecticide-treated bed net (ITN) ownership		
Yes	28	16.4
No	143	83.6
ITN utilization (applicable to only those who owned them)		
Never	5	17.9
1–2	9	32.1
3 times	7	25
> 4 times	7	25
Indoor residual spray (IRS) usage		
Yes	124	72.4
No	47	27.5
Sulfadoxine-pyrimethamine (IPT-SP) received dose in the clinic		
None	165	96.5
1 time	5	2.9
2 time	1	0.6

### Environmental and clinical characteristics related to malaria prevention and antenatal care services among pregnant women in Mogadishu, Somalia

Among the 171 individuals surveyed, a significant majority (97.7%) did not have access to free IPTp-SP, with only 2.3% confirming receipt of this intervention at no cost. A considerable proportion (76%) of respondents reported the presence of mosquitoes in their environment, whereas 24% did not. Regarding malaria education, only 8.2% of the women received any information, leaving 91.8% without such educational exposure. Additionally, only 14.6% of participants indicated that IPTp-SP was available at their health facility during their visit, underscoring a potential deficiency in access to preventive care (Table 3).

**Table 3:** Environmental and clinical characteristics related to malaria prevention and antenatal care services among pregnant women in Mogadishu, Somalia

Variables	Frequency	Percent (%)
IPTPsp Free availability in the clinic		
Yes	4	2.3
No	167	97.2
Household mosquito presence		
Yes	130	76.0
No	41	24.0
Malaria education in the clinic		
Yes	14	8.2
No	157	91.8

### Prevalence of asymptomatic Plasmodium species infection among pregnant women in Mogadishu, Somalia

This study revealed a 2.9% prevalence of asymptomatic *Plasmodium* infection in pregnant women, with *P. falciparum* accounting for all cases. The remaining 97.1% of participants tested negative.

### Factors associated with asymptomatic Plasmodium species infection among pregnant women in Mogadishu, Somalia

This study assessed 171 pregnant women with asymptomatic *Plasmodium* infection, considering various sociodemographic, obstetric, and malaria prevention factors. None of the factors analysed were significantly associated with asymptomatic malaria. Although women living in rural areas had a greater likelihood of infection than those living in urban areas (OR = 0.124; 95% CI 0.012–1.322), this relationship was not statistically significant (p = 0.165). Likewise, variables such as ANC attendance (OR = 4.30) and IPTp-SP availability (OR = 3.28) showed increased odds ratios, but the broad confidence intervals and non-significant p-values indicate that these results should be interpreted cautiously (Table 4).

**Table 4:** Association between socio-demographic, environmental, and clinical factors and asymptomatic *Plasmodium* infection among pregnant women in Mogadishu, Somalia

Variables	Asymptomatic *Plasmodium* status		OR (95%CI)	*P*-value
	Positive (%)	Negative (%)		
Age group				
18–24 years	1 (20.0%)	84 (50.6%)	0.24 (0.03–2.23)	0.368
> 24 years	4 (80.0%)	82 (49.4%)		
Educational status				
Non-Formal	3 (60.0%)	84 (50.6%)	1.464 (0.24–8.99)	1.000
Formal	2 (40.0%)	82 (49.4%)		
Residence				
Urban	4 (80.0%)	161 (97.0%)	0.124 (0.012–1.322)	0.165
Rural	1 (20.0%)	5 (3.0%)		
Occupational Status				
Employed	0 (0.0%)	12 (7.2%)	1.12 (0.06–21.5)	1.000
Un-employed	5 (100.0%)	154 (92.8%)		
Marital status				
Married	5 (100.0%)	150 (90.5%)	1.21 (0.06–22.8)	1.000
Widowed/Divorced	0 (0.0%)	16 (9.6%)		
Gestational age				
First trimester	2 (40.0%)	27 (16.3%)	–	0.320
Second trimester	1 (20.0%)	62 (37.3%)		
Third trimester	2 (40.0%)	77 (46.4%)		
Gravidity status				
One time	0 (0.0%)	43 (25.9%)	0.26 (0.01–4.76)	0.332
More than one time	5 (100.0%)	123 (74.1%)		
History of Malaria infection within previous year				
Yes	1 (20.0%)	62 (37.3%)	0.42 (0.05–3.83)	0.653
No	4 (80.0%)	104 (62.7%)		
ANC attending				
Yes	4 (80.0%)	80 (48.2%)	4.30 (0.47–39.3)	0.205
No	1 (20.0%)	86 (51.8%)		
ITNs ownership				
Yes	1 (20.0%)	27 (16.3%)	1.28 (0.14–11.96)	1.000
No	4 (80.0%)	139 (83.7%)		
ITN utilization				
Yes	0 (0.0%)	23 (85.2%)	0.06 (0.002–1.82)	0.179
No	1 (100.0%)	4 (14.8%)		
IRS usage				
Yes	5 (100.0%)	119 (71.7%)	4.37 (0.24–80.8)	0.324
No	0 (0.0%)	47 (28.3%)		
IPTPsp Free availability in the clinic				
Yes	0 (0.0%)	4 (2.4%)	3.28 (0.16–68.8)	1.000
No	5 (100.0%)	162 (97.6%)		
Household mosquito presence				
Yes	5 (100.0%)	125 (75.3%)	3.64 (0.20–67.6)	0.339
No	0 (0.0%)	41 (24.7%)		
Malaria Education in the clinic				
Yes	0 (0.0%)	14 (8.4%)	0.96 (0.05–18.4)	1.000
No	5 (100.0%)	152 (91.6%)		

## Discussion

This study assessed the prevalence of asymptomatic malaria and its associated risk factors among pregnant women. Both Giemsa-stained smears and rapid diagnostic testing revealed that 2.9% of these women had asymptomatic *Plasmodium* parasitaemia. These results agree with the findings of other studies conducted in the Oromia Region of Ethiopia (2.74%) [17], Bangladesh (2.3%) [18], North-Shoa, Ethiopia (3.4%) [19], the Merti District, Ethiopia (3.6%) [10], and Colombia (4.2%) [20]. However, this figure is lower than that reported in other studies conducted in the Republic of Congo (7%) [21], Ethiopia (7.2%) [22], Southern Laos (8.3%) [23], Nigeria (9.2%) [24], Burkina Faso (11%) [9], the Jawi District, Northwest Ethiopia (11.2%) [25], the Gurage Zone in Southern Ethiopia (15.2%) [26], the Majang Zone, Gambella Region, Southwest Ethiopia (15.3%) [8], Guinea (15.8%) [27], and in the West Guji Zone, Ethiopia (24.1%) [28]. Significant climatic changes, including variations in temperature, precipitation, and humidity, as well as patterns of human settlement and population movement, could also account for the observed differences in prevalence rates. The reduced occurrence of asymptomatic parasitaemia among pregnant women observed in this study might be attributed to the limited sample size, timing of the research during a period of low malaria transmission, and variations in malaria epidemiology across the study locations.

The results of this study indicated that *Plasmodium falciparum* was the sole species detected among the malaria-positive cases in the study area, representing 100% of the infections. This suggests that *P. falciparum* is the most prevalent *Plasmodium* species in the region. These results align with the WHO 2023 report that *P. falciparum* accounted for approximately 92% of all malaria cases in Somalia. The slight discrepancy in percentages could be due to variations in study design, sample size, and the timing or season of data collection; however, both sources clearly demonstrate the dominance of *P. falciparum* in the country [29]. Conversely, research conducted in other parts of East Africa has revealed a more varied distribution of *Plasmodium* species. For example, a study from the West Guji Zone in Ethiopia found *Plasmodium vivax* to be more common, accounting for 54.5% of cases, whereas *P. falciparum* was responsible for 45.5% [28]. Similarly, a study in the Majang Zone of the Gambella Region in Southwest Ethiopia reported *P. falciparum* as the predominant species, accounting for 55.4% of malaria cases [8].

These differences underscore regional variations in malaria epidemiology, which may be affected by ecological factors, vector species distributions, and local malaria control measures.

In this study, none of the sociodemographic, obstetric, or malaria prevention factors examined showed statistically significant associations with asymptomatic *Plasmodium* infection; however, several point estimates suggest potential trends worthy of interpretation. For instance, living in urban settings is associated with a lower chance of contracting malaria than residing in rural areas. Additionally, the utilization of insecticide-treated nets (ITNs) seemed to provide a degree of protection against the disease, whereas the impact of indoor residual spraying (IRS) did not completely match the anticipated patterns of malaria risk. These results add to the expanding body of evidence highlighting the intricate relationship between geographic, environmental, and behavioural factors that influence malaria transmission dynamics. The protective effect of living in urban areas aligns with the results of several studies conducted in Ethiopia. For instance, a study in the Jawi District of Northwest Ethiopia found that people in rural regions had a 4.51 times greater chance of malaria infection, reinforcing the connection between rural environments and increased transmission risk [25].

Our findings on ITN use are consistent those of studies conducted in other regions of Ethiopia. In the North-Shoa, ITN usage was a significant predictor of a lower malaria infection risk [19]. Similarly, research from the Majang Zone in the Gambella Region identified ITN non-use as a strong independent risk factor [8]. In the Boset District of Oromia, lack of ITN use was also significantly associated with infection [17], whereas in the West Guji Zone, ITNs were not identified as a key risk factor for asymptomatic malaria during pregnancy [28]. Supporting these findings, a study in the Jawi District of Northwest Ethiopia likewise reported that non-use of ITNs was strongly linked to an elevated infection risk [25]. Collectively, these studies highlight the consistent and substantial protective effect of ITN use across diverse ecological and geographic contexts, particularly in vulnerable populations such as pregnant women.

The current study found no link between IRS and reduced malaria risk, which is consistent with the North-Shoa study [19]. However, this contrasts with findings from West Guji, Majang, and Jawi districts, where IRS was a key protective measure [8, 25, 28]. This difference might be due to several factors specific to the context, such as the timing and frequency of IRS application, insecticide resistance, or differences in housing structures that affect IRS efficacy. It also highlights the importance of closely examining the operational quality and reach of the IRS programs in the study areas. In this study, no significant association was found between ANC attendance and asymptomatic malaria. Similarly, the West Guji study also reported no significant association between ANC follow-up and malaria infection risk [28]. However, a previous study in the Majang Zone reported that fewer ANC follow-up visits were a strong predictor of infection later in pregnancy, particularly in asymptomatic cases [8]. The observed differences might be due to regional variations in the intensity of malaria transmission, the availability of healthcare services, and the quality and timing of antenatal care (ANC) visits. No significant correlation was observed between the gestational age and infection rate. However, the observation of higher infection rates during the first and third trimesters aligns with research from Kwale County, Kenya, which found that a gestational age ≥ 16 weeks was associated with a heightened risk of asymptomatic malaria infection during pregnancy [30]. This is in contrast to the findings of a study conducted in the Sherkole district of Ethiopia, which reported a statistically significant association between gestational age and malaria infection. The study found that women in the first and second trimesters had substantially higher odds of malaria infection than those in the third trimester [31].

Although all infections were observed in women who had been pregnant more than once, the number of pregnancies was not statistically significant. Similarly, the Kwale County, Kenya study reported no significant association between gravidity and asymptomatic malaria [30]. In contrast, studies from the Jawi District, the Republic of Congo, and a national meta-analysis of Ethiopian studies found that being pregnant for the first time was a significant risk factor for asymptomatic infection [22, 25, 32]. A history of malaria within the previous year was also not significantly associated with infection, which contrasts with the results from the West Guji and Majang zones, where a previous infection greatly increased the likelihood of asymptomatic malaria [8, 28].

The availability of IPTp-SP in clinics was not significantly associated with the malaria status, although the sample size was insufficient to draw definitive conclusions. Conversely, data from the Republic of Congo indicated that IPTp-SP was associated with a reduced prevalence of asymptomatic *P. falciparum* infections [21]. This discrepancy may reflect differences in IPTp-SP coverage, adherence, or local transmission dynamics, underscoring the need for larger, context-specific evaluations of IPTp-SP effectiveness. In this study, no sociodemographic factors were significantly associated with asymptomatic malaria. Younger women (18–24 years) showed lower odds of infection, consistent with the findings from the Majang Zone [8]. Educational and occupational status also showed no notable associations, in line with the results from West Guji and Jawi [25, 28]. These results were further supported by data from the Republic of Congo, which also found no significant association between age or educational status and asymptomatic malaria [21].

In this study, malaria education was not significantly associated with the infection status. Although all positive cases were observed among women who had not received malaria education, the limited sample size precluded the drawing of definitive conclusions. This finding contrasts with the results from the Boset District in Oromia and the Sherkole District in Benishangul Gumuz, Ethiopia, where the absence of consultation and health education during antenatal care (ANC) was significantly correlated with increased odds of asymptomatic malaria [17, 31]. Similarly, a systematic review from Ethiopia identified a lack of malaria prevention education as a strong predictor of infection among pregnant women [33]. These discrepancies may be attributed to variations in sample size, quality, timing of education, or the overall intensity of malaria transmission. One drawback of this study was its cross-sectional nature, which limited the ability to establish causal links between the variables and its findings may not be applicable to other regions. Additionally, data collection occurred during the dry season, which is typically linked to a low rate of malaria transmission. This seasonal aspect might have resulted in an underestimation of the actual prevalence of asymptomatic malaria the study group.

## Conclusions

This study found a 2.9% prevalence of asymptomatic malaria among pregnant women, with Plasmodium falciparum being the sole species identified. No significant statistical links were found between infection and sociodemographic, obstetric, or malaria prevention factors, although trends indicated a reduced risk in urban dwellers and those using ITNs. The absence of significant associations with factors such as IRS, ANC attendance, and malaria education differs from the results in other Ethiopian regions, indicating the impact of local transmission patterns, intervention quality, and sample size. These findings emphasize the complexity of asymptomatic malaria during pregnancy and highlight the necessity for prevention strategies tailored to specific contexts as well as further research with larger sample sizes to inform targeted interventions in this area. Future research should use highly sensitive molecular diagnostic techniques to accurately determine the true extent of asymptomatic malaria and to guide effective public health measures.

## Data Availability

The data upon which the results are based are available from the corresponding author on request.

## Abbreviations

ANC: Antenatal care
anti-HRP-II: Anti-Histidine-Rich Protein II
anti-LDH: Anti-Lactate Dehydrogenase
API: Asymptomatic infection
EDTA: Ethylenediaminetetraacetic acid
IPT-SP: Sulfadoxine-pyrimethamine
IRS: Indoor residual spray
ITN: Insecticide-treated bed net
RDTs: Rapid diagnostic tests
WHO: World Health Organization
